# Impact of autoimmune diseases on outcome of patients with early breast cancer

**DOI:** 10.18632/oncotarget.9966

**Published:** 2016-06-13

**Authors:** Carmen Criscitiello, Vincenzo Bagnardi, Angela Esposito, Lucia Gelao, Barbara Santillo, Giulia Viale, Nicole Rotmensz, Aron Goldhirsch, Giuseppe Curigliano

**Affiliations:** ^1^ Division of Experimental Therapeutics, Istituto Europeo di Oncologia, Milano, Italy; ^2^ Division of Epidemiology and Biostatistics, Istituto Europeo di Oncologia, Milano, Italy; ^3^ Department of Statistics and Quantitative Methods, University of Milan-Bicocca, Milano, Italy; ^4^ Breast Cancer Program, Istituto Europeo di Oncologia, Milano, Italy

**Keywords:** autoimmunity, early breast cancer, endocrine therapy, clinical outcome, immunology

## Abstract

Our aim was to analyze the impact of a concurrent autoimmune disease on outcome of patients with early breast cancer. We reviewed medical charts of patients with a diagnosis of autoimmune diseases (AD) among a population of 17.153 cases. We categorized ADs as endocrine, rheumatic, systemic, neurological diseases and vasculitis. For each patient in the study group, we matched 2 patients. The events to determine overall survival (OS) and disease free survival (DFS) were identified from follow-up data. We identified 279 (1.62%) patients with early breast cancer and concurrent ADs. The median follow-up was 7.0 years. The 10-year OS rate was 86% (95% CI, 80% to 91%) in the study group and 90% (95% CI, 86% to 93%) for the control group (*p* = 0.011). In patients with ER positive/HER2 negative subtype a worse OS was observed in the study group when compared to the control group (*p* = 0.0046); this difference remained statistically significant when the analysis was restricted to breast cancer mortality (*p* = 0.045). The 10-year DFS rate was 69% (95% CI, 61% to 76%) in the study group and 72% (95% CI, 66% to 77%) for the control group (*p* = 0.22). Autoimmunity at diagnosis of early breast cancer is associated with worse survival.

## INTRODUCTION

Autoimmune diseases (ADs) affect approximately 7–10% of individuals living in the western countries, representing a significant cause of morbidity, chronic disability and health-care burden [1]. A population-based cohort study of more than 20 000 1-year survivors of childhood cancer showed a significant, 1.4-fold increase in risk for a hospital contact for an autoimmune disease over that of the background population [2]. One hypothesis to explain the increase in risk for several autoimmune diseases is that persistent immune abnormalities after treatment with chemotherapy and the cancer itself predispose to the development of autoantibodies, which are central to the pathogenesis of many autoimmune diseases. On the other hand limited evidences are available on the outcome of patients affected by ADs who develop cancer. ADs are associated with activation of auto-reactive T and B lymphocytes, down-regulation of T regulators cell and release of pro-inflammatory cytokines leading to B and T cells tolerance loss. Natural and inducible CD4+ CD25+ Forkhead box P3 (FOXP3)+ regulatory T cells (Tregs) play a key role in maintaining immune self-tolerance, as they inhibit the activation and expansion of autoreactive immune clones; hence, their absence or functional defects favor the development of various autoimmune diseases [3]. Moreover Tregs are involved in the inhibition of effector functions in both inflammation and cancer; it has been shown that the presence of CD4+CD25+FOXP3+ regulatory T cells in cancer tissues is associated with mechanisms of tumor immune escape and worse prognosis [4, 5]. The expression of the transcription regulator FOXP3 is necessary for Tregs suppressor function [6]. Also, loss or downregulation of FOXP3 lead to the acquisition of effector T cell properties [7]. T helper 17 cells (Th17), producing IL-17A, IL-17F, IL-21, IL-22 and IL- 26, are important co-regulators. The balance between T reg and Th17 cells regulates TH1/TH2 shift in ADs [8]. However, little is known on the balance between Treg and Th17 cells in patients with cancer and on the active role played by Th 17 in anti-tumor immunity [9]. IL17 promotes host defenses against bacterial infection [10] and elevated levels of these cytokines have been associated to inflammatory systemic diseases such as rheumatoid arthritis (RA) and systemic lupus erythematosus (SEL) [11]. The presence of IL 17 in tumor tissues seems to be protective, due to its ability to activate CD8+ cell priming; however, its role is still controversial [12, 13]. Other important guardians of immune self-tolerance are dendritic cells (DC), which are involved in either initiating (mature DC) or silencing T cell responses (immature DCs- iDCs) [14]. The correlation between some ADs such as Sjogren syndrome, RA, SEL and lymphoproliferative malignancies is well established [15], as well as the association between dermatomyositis and the development of solid tumors [16]. Several studies have suggested that ADs may differently influence the risk of breast cancer (BC), either increasing it or reducing it. A recent review of the literature has highlighted the positive epidemiologic correlation between ulcerative colitis, psoriasis, Graves disease, multiple sclerosis and the development of BC [17–20]. Moreover, some therapies used in autoimmune diseases, such as tumor necrosis factor (TNF) blockers, eventually interact with the development of tumors, even though clinical studies on this topic showed discordant results. It is worth mentioning here that new immunomodulatory antibodies targeting Cytotoxic T-Lymphocyte Antigen 4 (CTLA-4) and Programmed Death-1 (PD-1), currently under investigation for cancer treatment, have important immune system related adverse events [21, 22]. The aim of our study was to investigate whether clinical features and tumor characteristics differ in BC patients with and without concurrent autoimmune comorbidities. We also assessed whether a concurrent ADs impact on BC outcome in terms of both disease free survival (DFS) and overall survival (OS).

## RESULTS

**Figure 1:**
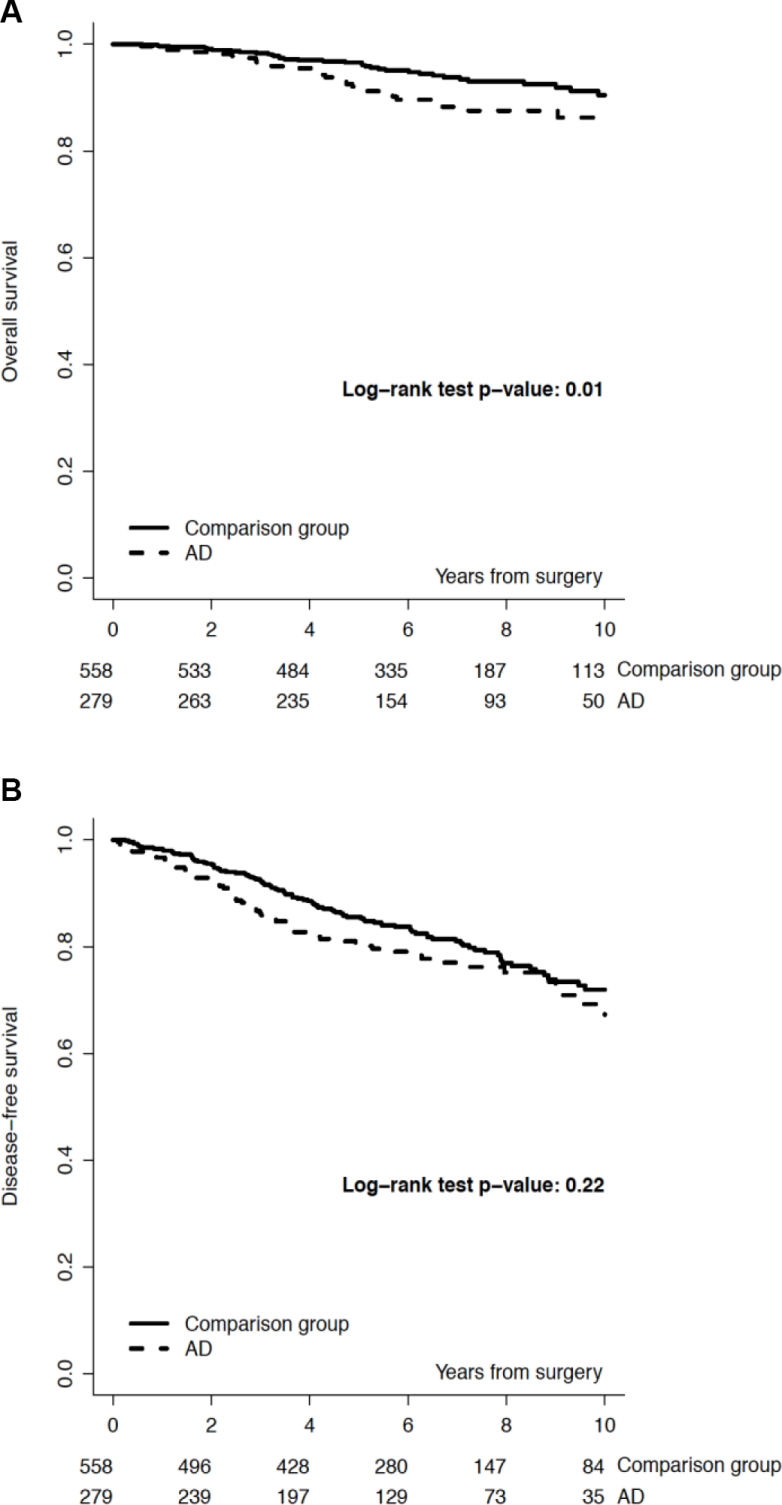
Overall survival (Panel A) and Disease-free survival (Panel B), by study group

**Figure 2:**
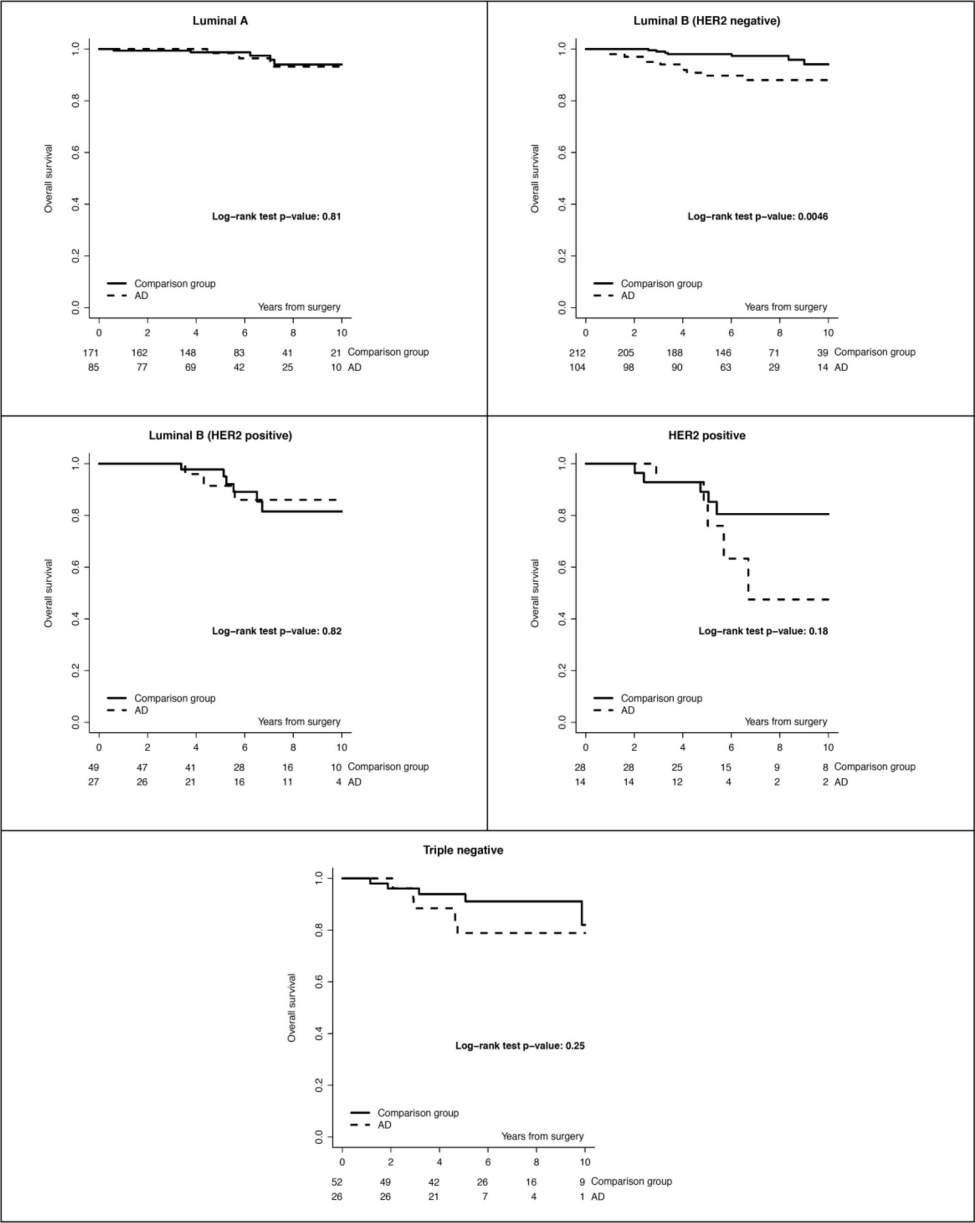
Overall survival by study group and surrogate intrinsic subtype

**Figure 3:**
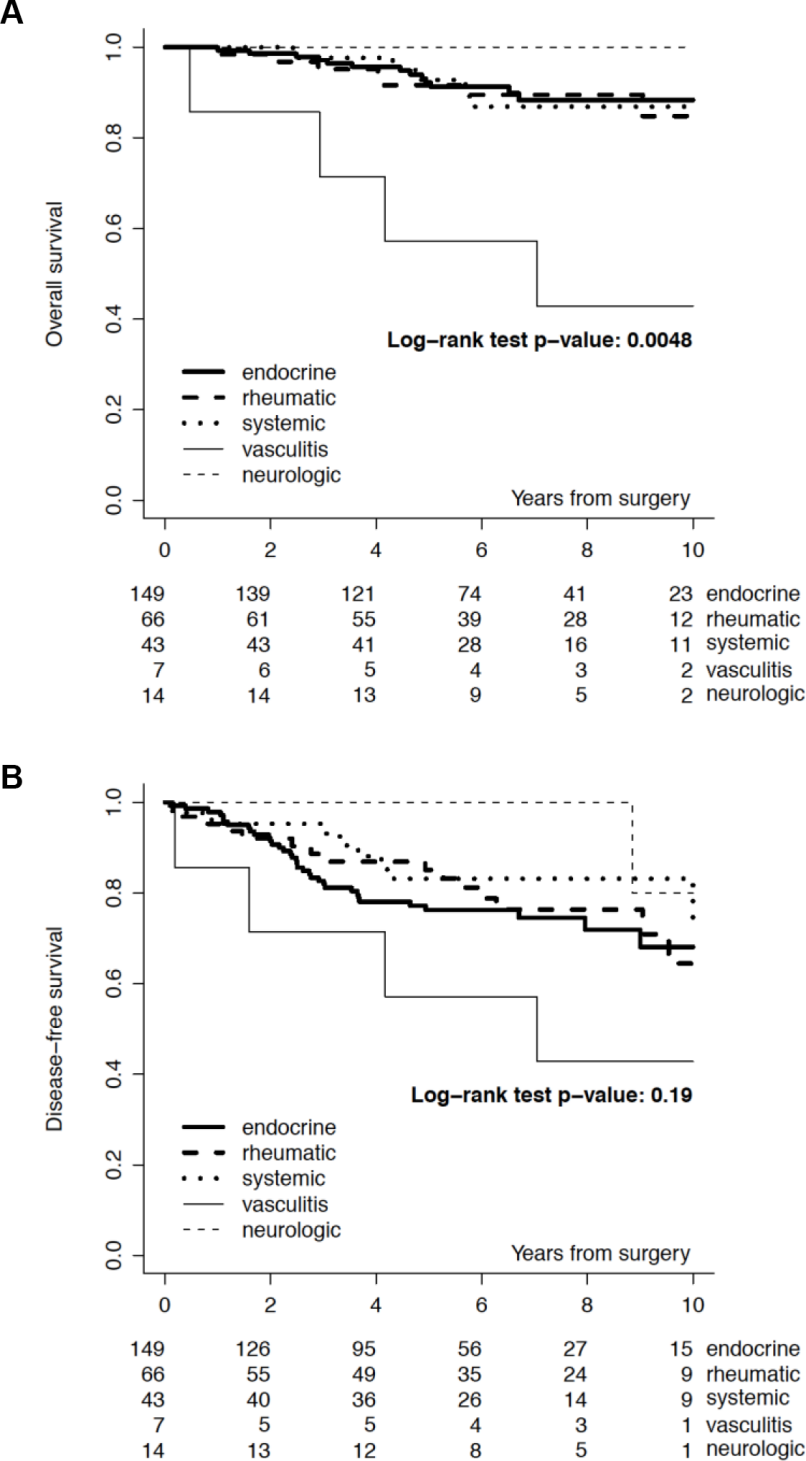
Overall survival (Panel A) and Disease-free survival (Panel B), by type of autoimmune disease

We identified 279 (1,6%) patients with early breast cancer and concurrent ADs out of 17.153 cases screened (Table 1). We classified 149 (53.4%) patients with autoimmune endocrine diseases (i.e. thyroiditis, Basedow disease, type I diabetes), 66 (23.7%) with rheumatic diseases, 43 (15.4%) with systemic autoimmune diseases (i.e. SEL, scleroderma disease, Sjogren syndrome, sclerotic cholangitis), 14 (5%) with neurologic autoimmune like diseases (i.e. miastenia gravis, sclerosis multiple, Guillain-Barré syndrome) and 7 (2.5%) with vasculitis. Almost all patients included in the study group received an adjuvant therapy: 147 (52.7%) an endocrine therapy alone, 39 (14%) a chemotherapy alone and 72 (25.2%) received chemotherapy and endocrine therapy; 21 (7.5%) of patients received no treatment. Almost all patients included in the study group had an estrogen receptor (ER) positive/HER2 negative breast cancer (67.8%), 27 (9.7%) patients had a ER positive/ HER2 positive breast cancer, 14 (5%) had an HER2 positive/ER and PgR negative and 26 (8.2%) had a triple negative breast cancer. There was no significant correlation between age, menopausal status, histology, surrogate intrinsic subtypes and incidence of autoimmune disease. Baseline demographic, clinical, and pathologic characteristics and local and systemic treatments of breast cancer patients with autoimmune disease (AD) and a matched cohort of breast cancer patients without AD are reported in Table 1. The median follow-up was 7.0 years. We recorded the incidence of events in the study and control group. In the DFS analysis we observed 66 (23.7%) events in the ADs group and 117 (21%) in the control group. We recorded 25 (9.0%) locoregional events and 22 (7.9%) distant events in the ADs group and 38 (6.8 %) locoregional events and 42 (7.5%) distant events in the control group. The 5-year DFS rate was 80% (95% CI, 75% to 85%) in the study group and 86% (95% CI, 82% to 88%) for the control group. The 10-year DFS rate was 69% (95% CI, 61% to 76%) in the study group and 72% (95% CI, 66% to 77%) for the control group (*p* = 0.22). In the OS analysis we observed 32 (11.5%) deaths in the ADs group and 36 (6.4%) in the control group (*p* = 0.01). The causes of deaths in the study (AD) group were breast cancer related in 22 (7.9%) patients, related to a second primary in 2 (0.7%) patients and related to other disease in 8 (2.9%) patients. Causes of deaths in the control group were breast cancer related in 28 (5.0%) patients, related to a second primary in 2 (0.4%) patients and related to other disease in 6 (1.1%) patients. The 5-year OS rate was 92% (95% CI, 87% to 94%) in the study group and 97% (95% CI, 95% to 98%) for the control group. The 10-year OS rate was 86% (95% CI, 80% to 91%) in the study group and 90% (95% CI, 86% to 93%) for the control group (*p* = 0.0112). Figure 1, panel A and panel B, reports OS and DFS by study group. When looking at OS by surrogate intrinsic molecular subtypes, a statistically significant difference between study and control group was observed only in the ER positive/HER-2 negative subtype (p 0.0046, Figure 2); this difference remained statistically significant when the analysis was restricted to breast cancer mortality (9% breast cancer mortality at 10-yr in the study group vs 6% in the control group, *p* = 0.045). In terms of DFS, no significant differences were observed (p 0.22). The absence of differences in DFS remained in the surrogate intrinsic molecular subtypes analysis. Finally, we assessed a correlation between OS and specific type of ADs. We observed a definitely worse outcome for patients affected with vasculitis, although there were very few patients (Figure 3).

**Table 1:** Baseline demographic, clinical, and pathologic characteristics and local and systemic treatments of breast cancer patients with autoimmune disease (AD) and a matched cohort of breast cancer patients without AD

	Patients with autoimmune disease (AD) *N* (%)	Comparison group *N* (%)	*p*-value
All patients	279 (100)	558 (100)	
**Type of AD**			
endocrine	149 (53.4)	–	–
rheumatic	66 (23.7)		
systemic	43 (15.4)		
vasculitis	7 (2.5)		
neurologic	14 (5.0)		
**Year of surgery**			matching variable
< 1998	9 (3.2)	14 (2.5)	
1998–2001	48 (17.2)	97 (17.4)	
2002–2004	50 (17.9)	106 (19)	
2005–2007	97 (34.8)	199 (35.7)	
2008–2010	75 (26.9)	142 (25.4)	
**Age at surgery**			matching variable
< 35	9 (3.2)	15 (2.7)	
35–50	109 (39.1)	220 (39.4)	
51–65	116 (41.6)	230 (41.2)	
> 65	45 (16.1)	93 (16.7)	
**Menopausal status**			matching variable
Peri-Pre	125 (44.8)	250 (44.8)	
Post	154 (55.2)	308 (55.2)	
**Histology**			0.58
Ductal	225 (80.6)	437 (78.3)	
Lobular	25 (9)	63 (11.3)	
Mixed/Other	29 (10.4)	58 (10.4)	
**Tumor size (pT)**			0.46
pT1	179 (64.2)	374 (67.1)	
pT2	91 (32.6)	164 (29.4)	
pT3/pT4	9 (3.2)	20 (3.6)	
**No. of positive lymph nodes**			matching variable
pNx	7 (2.5)	14 (2.5)	
None	154 (55.2)	308 (55.2)	
1–3	75 (26.9)	150 (26.9)	
4+	43 (15.4)	86 (15.4)	
**Grade**			0.16
Unknown	12 (4.3)	15 (2.7)	
1–2	168 (60.2)	356 (63.8)	
3	99 (35.5)	187 (33.5)	
**Surrogate intrinsic subtype**			matching variable
Luminal A	85 (30.5)	171 (30.6)	
Luminal B (Her2 negative)	104 (37.3)	212 (38)	
Luminal B (Her2 positive)	27 (9.7)	49 (8.8)	
Her2 positive	14 (5)	28 (5)	
Triple Negative	26 (9.3)	52 (9.3)	
Missing	23 (8.2)	46 (8.2)	
**Perivascular Invasion**			0.71
Absent	203 (72.8)	391 (70.1)	
Focal	46 (16.5)	103 (18.5)	
Diffuse	30 (10.8)	64 (11.5)	
**Surgery**			0.82
Conservative	213 (76.3)	430 (77.1)	
Mastectomy	66 (23.7)	128 (22.9)	
**Radiotherapy**			0.24
No	54 (19.4)	83 (14.9)	
Yes-External	143 (51.3)	309 (55.4)	
Yes-Intraoperative	82 (29.4)	166 (29.7)	
**Systemic adjuvant therapy**			0.39
Nil	21 (7.5)	27 (4.8)	
Endocrine therapy alone	147 (52.7)	310 (55.6)	
Chemotherapy alone	39 (14)	70 (12.5)	
Chemotherapy and endocrine therapy	72 (25.8)	151 (27.1)	

## DISCUSSION

The aim of this study was to analyze the impact of a concurrent autoimmune disease on clinical outcomes of patients with early breast cancer. The clinical and pathological features of the two cohorts were well-balanced. According to the results of our retrospective analysis ADs may impact on outcome of patients with early BC, thus suggesting that comorbidities may impact on therapeutic decision and treatment delivery, tolerability and compliance. Reluctance to prescribe systemic treatments was due to the complexity of evaluation for these patients and to the burden of symptoms related to concurrent ADs. Taking into account the data from the current study and given the climate of uncertainty regarding optimal treatment, we believe that physicians decided to individualize care on the basis of biologic characteristics, comorbidity, functional status, and patient preferences. We also evaluated whether ADs may affect BC outcome and response to treatment. In this series, the presence of ADs seems to negatively influence BC outcome, particularly OS. From survival analyses, we noticed that statistically significant better OS (*p*-value = 0.0112) was reported for BC patients with no ADs as compared to patients with BC and ADs, both at 5 (97% vs 92%) and 10 years (90% vs 86%), regardless of type of ADs. Mortality rate was higher in patients with ADs (11.5% vs 6.4% in controls), and deaths were mainly due to BC in both cohorts. However, when OS data were analyzed according to tumor subtype, a statistical significant difference was observed only in the Luminal B/HER2-negative subtype. The potential explanation for this observation may be related to the poor compliance of patients to treatment with endocrine therapy. These agents may exacerbate arthralgia and other symptoms frequently associated to ADs. To determine appropriate intervention in this group of patients, it is therefore essential to perform a comprehensive baseline evaluation of musculoskeletal complaints. Our results are in conflict with those by Einefors et al, who showed improved BC outcome in patients with ADs [23]. Indeed, among the 1705 BC patients included in that analysis, those with coexisting AD (125 patients) had a not significant trend towards better OS, adjusted for age at diagnosis and TN stage (HR 0.91; CI 0.72–1.14). The benefit of the concurrent presence of AD or hypersensitivity disease (asthma or allergies) resulted to be higher for patients with ER-negative BC, who achieve a significant improvement of OS irrespective of menopausal status. This study analyzed BC outcome in the cohort through a multivariate analysis, whereas in our study the two cohorts were matched according to multiple parameters. ADs might influence BC outcome, like any comorbidity, for example by worsening the general health status of patients or by condition therapeutic options. Moreover, ADs could interfere with anti-cancer therapy, particularly with immunotherapeutics, such as trastuzumab, whose anti-cancer effect partly relies on antibody-dependent cell-mediated cytotoxicity. An improved outcome was observed in BC patients with antithyroid peroxidase antibodies; moreover antithyroid peroxidase antibody positivity seems to be associated with a lower incidence of metastases in BC patients [24]. Other authors suggested that this could be due to the presence of a BC/thyroid shared antigens, probably antithyroid peroxidase antibody itself, that is weakly expressed in BC tissue and is responsible for the immunoreactivity showed in laboratory by antithyroid peroxidase antibodies positive patients serum against cancer tissue cells [25]. In another study was assessed the relationship between antiestrogen therapy in women with breast cancer and risk of autoimmune disease [26]. A national database was used to assess the incidence of systemic lupus erythematosus (SLE) and rheumatoid arthritis (RA) following treatment with selective estrogen receptor modulators (SERM) or aromatase inhibitors (AI) in women with breast cancer. The total number of patients in our study was 190,620. Authors observed highly significant, cumulative dose-dependent increased OR of incidence of both SLE and RA following treatment with SERM (*p* < 0.0001) [26]. The odds of developing RA were also increased following AI therapy (*p* < 0.001), but there was a trend for reduced odds of SLE, though this trend did not attain statistical significance (*p* = 0.070 for 2-11 months of treatment and *p* = 0.254 for 12+ months of treatment). In this analysis antiestrogen agents may have an important effect on risk of autoimmune disease [26]. Our study have several limitations. We used diagnosis of autoimmune diseases as a parameter to be correlated with the outcome of early breast cancer. The analysis included a variety of different diseases such ad autoimmune endocrine diseases, rheumatic diseases, systemic autoimmune diseases, and others. Given that all these diseases are characterized by different autoimmune mechanisms and come with different treatment approaches, we believe that this generalized approach has clear limitations. Another limitation is related to the different treatment regimens of the autoimmune diseases that should be taken into account when evaluating our data. For almost all patients we don't have appropriate information on type treatment. Assessment of clinical outcome of the autoimmune disease activity would be more appropriate to elucidate potential causal interconnections. The results of our analysis confirm the relevance of ADs on the outcome of patients with early BC. In our study, autoimmune comorbidities in particular correlate significantly with a negative OS outcome in estrogen receptor positive disease. The presence of ADs should be considered when deciding on therapeutic strategies in patients with early BC. Further longitudinal studies are needed in order to confirm these findings, including also other comorbidity patterns, and to investigate the relationship between comorbidities, disease course and response to treatment.

## MATERIALS AND METHODS

We retrospectively retrieved from our Institutional prospective Breast Cancer Data Base - 279 consecutive patients with early breast cancer and concurrent autoimmune disease (AD), who underwent surgery at our Institution between 1994 and 2009. Patients with previous history of cancer, bilateral metastatic breast cancer were excluded, as well as patients who previously received neoadjuvant therapy. We defined and categorized ADs as autoimmune endocrine diseases (i.e. thyroiditis, Basedow disease, type I diabetes), RA, systemic rheumatic diseases (i.e. RA), systemic autoimmune diseases (i.e. SEL, scleroderma disease, Sjogren syndrome, sclerotic cholangitis), neurologic autoimmune like diseases (i.e. miastenia gravis, sclerosis multiple, Guillain-Barré syndrome) and vasculitis. A comparison group of 558 patients was obtained by randomly selecting, for each patient in the study group, two patients matched for year of diagnosis (within 2 years), age (within 5 years), menopausal status, number of positive lymph nodes and surrogate intrinsic subtype. Demographic data, clinical and biological features, treatment data for BC patients with ADs and for the matched group of BC patients without ADs were retrieved from the data base. Immunohistochemical staining for estrogen receptor (ER), PgR, HER2 protein and Ki-67 antigen was performed with consecutive tissue sections from the same tumor blocks. The following primary antibodies were used: the monoclonal antibody (mAb) against ER (clone 1D5, 1:100 dilution; Dako, Glostrup, Denmark), the mAb against PgR (clone 1A6, 1:800 dilution; Dako), the MIB-1 mAb against Ki-67 antigen (1:100 dilution; Dako) and the polyclonal antibody against HER2 protein (1:3,200 dilution; Dako). Only nuclear reactivity was taken into account for ER, PgR and Ki-67, irrespective of the staining intensity, whereas only intense and complete membrane staining in >10% of the tumor cells were considered HER2 overexpression (3+). In addition, fluorescence *in situ* hybridization assays were performed for the final determination of HER2 status for tumors with 2+ immunoreactivity. Median follow-up was 7 years. OS, DFS and causes of death have been assessed and by surrogate intrinsic subtype. We also assessed any associations between type of ADs and overall survival.

### Statistical analysis

The chi-square test was used to assess differences between the study and the control group in the distribution of prognostic variables and treatment approaches. The main endpoints were overall survival (OS) and disease-free survival (DFS). OS was defined as the length of time from the date of surgery to death from any cause. DFS was defined as the length of time from the date of surgery to events such as relapse (including ipsilateral breast recurrence), appearance of a second primary cancer (including contralateral breast cancer), or death, whichever occurred first. For survivors, OS and DFS were censored at the last follow-up visit. The OS and DFS distributions were estimated by using the Kaplan-Meier method, and the log-rank test was used to test differences in survival distributions between study and control group.
